# Placental growth disorders and perinatal adverse outcomes in Brazilian HIV-infected pregnant women

**DOI:** 10.1371/journal.pone.0231938

**Published:** 2020-04-30

**Authors:** Helena Lucia Barroso dos Reis, Neide Aparecida Tosato Boldrini, Ana Fernanda Ribeiro Rangel, Vinicius Felipe Barros, Paulo Roberto Merçon de Vargas, Angélica Espinosa Miranda

**Affiliations:** 1 Post-Graduate Program of Infectious Diseases, Federal University of Espírito Santo, Vitória, Espírito Santo State, Brazil; 2 Department of Gynecology and Obstetrics, Federal University of Espírito Santo, Vitória, Espírito Santo State, Brazil; 3 Department of Pathology, Pathology Laboratory of Cassiano Antonio Moraes University Hospital, Federal University of Espírito Santo, Vitória, Espírito Santo State, Brazil; Ospedale dei Bambini Vittore Buzzi, ITALY

## Abstract

Fetal and placental growth disorders are common in maternal human immunodeficiency virus (HIV) infection and can be attributed to both the infection and comorbidities not associated with HIV. We describe placental growth disorders and adverse reproductive outcomes in HIV-infected pregnant women whose delivery occurred between 2001–2014 in Vitoria, Brazil. Cases with gestational age (GA) ≥ than 22 weeks validated by ultrasonography, with placental and fetal weight dimensions at birth, were studied. Outcomes were summarized as proportions of small (SGA), appropriate (AGA), and large (LGA) for GA when the z-score values were below -1.28, between -1.28 and +1.28, or above +1.28, respectively. Of 187 fetal attachment requisitions, 122(65.2%) women and their newborns participated in the study. The median maternal age was 28 years and 81(66.4%) underwent ≥ 6 prenatal visits. A total of 81(66.4%) were diagnosed before current pregnancy; 68(55.7%) exhibited criteria for acquired immunodeficiency syndrome (AIDS); 64(52.4%) had detectable viral load; 25(20.5%) cases presented SGA placental weight and 6(4.9%) SGA placental thickness. SGA placental area was observed in 41(33.6%) cases, and among the SGA placental weight cases 12(48%) were also SGA fetal weight. Preterm birth (PTB) occurred in 15.6%(19/122) of cases; perinatal death in 4.1%(5/122) and HIV vertical transmission in 6 of 122 (4.9%). Women, ≥36 years old, were 5.7 times more likely to have PTB than those under 36. Also, patients with AIDS-defining criteria were 3.7 times more likely to have PTB. Prenatal care was inversely associated with PTB. Statistically significant associations were observed between AGA placental area and Protease Inhibitor usage and between SGA placental weight and SGA area. We found a prevalence of placental growth disorders in HIV-infected pregnant women and values higher than international reference values. The restriction of placental growth was a common disorder, possibly attributed to virus effects or a combination of antiretroviral regimens.

## Introduction

The placenta is the organ of fetal adaptation to the maternal environment that is responsible for mechanical protection, nutrition, hormone production, gas exchange, hydro electrolytic control, and elimination of fetal excreta [1]. A placental anatomopathological examination is fundamental for evaluating the development of fetal annexes and identifying possible intrauterine and postnatal growth disorders, as well as diseases in adulthood [2,3]. Placental weight is a strong predictor of infant weight at birth and is commonly used as a summary of total organ growth, presumably reflecting maternal support, efficiency, and the functional adaptive capacity of the placenta [4]. However, it has been shown that weight results from two distinct growths: the lateral expansion of the chorionic plate (area) and the vertical (thickness), as well as the arborization of the villi and the vascular surface of maternal-fetal exchange [5,6].

In addition to placental weight, an assessment of placental area and thickness can be made during the second-trimester ultrasound examination, considering that a small placenta would be an indication of poor gestational prognosis and a predictor of preeclampsia [7] or perinatal morbidity. Moreover, previous studies have shown that placental volume estimated by ultrasonographic evaluation in the second trimester can predict both placental and birth weight [8].

In HIV-infected pregnant women using antiretroviral therapy (ART), an evaluation of fetal and placental growth enables the elucidation of several important factors that interfere with perinatal morbidity and mortality [9,10]. However, a common difficulty in previous studies is the need to control outcomes not only for comorbidities that affect growth and are prevalent in general gestations and in HIV-infection status (hypertensive disorders, smoking, diabetes, illicit drugs, fetal malformation, congenital syphilis and maternal body mass index (BMI) disorders) but also for the use of highly active antiretroviral therapy (HAART) [9,10]. In the literature related to adverse perinatal outcomes, conflicting data have been presented regarding the occurrence of restricted growth and preterm births in pregnant women infected with HIV under antiretroviral schemes with and without protease inhibitors (PI) before and after conception [10–14]. In this context, and considering the evidence that the postnatal growth of the children of HIV-infected women is often delayed or fails to meet expected values even without the occurrence of vertical transmission (VT) of the virus [15],new studies about fetal and placental growth in HIV-infected pregnant women are justified. Therefore, the objective of this study is to describe the placental growth disorders and adverse reproductive outcomes in HIV-infected pregnant women.

## Materials and methods

This descriptive study was conducted with data collected from medical and institutional records of HIV-infected pregnant women on fetal annexes and concepts present at the Laboratory of Pathology of the University Hospital in Vitoria, and whose terminations occurred between November 2001 and November 2014. This hospital has a reference maternity hospital for pregnant women with high obstetric risk in the state of Espírito Santo, Brazil. The inclusion criteria were maternal diagnosis of HIV infection, gestational age (GA) confirmed by ultrasonography equal to or greater than 154 days (22 weeks post-menstrual period; PMP), and fetal attachments with anatomopathological examination performed at the pathology unit. Twin pregnancies were excluded because of the difficulty in determining the chorion partition. GA was based on the date of the last menstruation period (LMP). In cases of compatibility between GA and the fetal biometry (evaluated in the ultrasound examination conducted up to 20 GA) [16]—and for cases with differences greater than seven days between the calculated GA by LMP and fetal biometry or with unknown LMP—the GA calculation was based on echographic parameters [17]. The immunological status of pregnant women was classified as HIV or AIDS using the criteria adapted from the Centers for Disease Control and Prevention (CDC), the Rio de Janeiro-Caracas criteria (1992), or the documented medical diagnosis of AIDS. The CD4 + T lymphocyte (TL) values and viral load levels were obtained from the medical records, and the diagnoses of maternal and child infection followed the recommendations of Brazil’s Ministry of Health. The VT case was confirmed with two positive viral loads or reactive serology of antibodies -HIV (ELISA) after 18 months of age [18].

Regarding ART, the treatment regimen considered as a variable for the analysis was the use or non-use of high-potency ART and use or non-use of PI. They were considered “used” when the medication was taken for at least five consecutive weeks. The timing of HIV diagnosis was categorized before and during gestation and at the time of delivery. As a reference value for birthweight, the local average weight of 3,247 g for live births in the state of Espírito Santo was used, according to data from the Live Birth Information System (SINASC) [19].

In this study, low birthweight was categorized as being less than 2,500 g., and births before the 37th week were considered preterm. The spontaneous preterm birth was one whose vaginal or cesarean delivery occurred after premature rupture of membranes, or with a diagnosis of preterm labor without other conditions. Iatrogenic or indicated preterm birth was considered when presenting a maternal indication for delivery, such as preeclampsia; or a fetal indication, such as fetal distress or intrauterine growth restriction; and the intervention was performed by cesarean section without labor, or by induction of labor [20].

Examinations of specimens from fetal annexes and perinatal death were performed according to standard procedures in the pathology unit, and they were examined macroscopically after fixation in a 10% formaldehyde solution. The placenta weight was measured without the umbilical cord and extra placental membranes [21], and the placenta area was calculated by tracing and dot-counting planimetry [22]. The placenta tracing is a drawing on a plastic sheet superimposed on the chorion plate of the contour and anatomical landmarks (e.g., insertion site of the umbilical cord, the inferior pole of the placenta, and any visible lesions on the fetal and maternal faces). The point scores [22,23] were added to a copy of the paper trace using the quadratic graticule of 1, 4, and 16 points, with areas of 1.0, 4.0, and 16.0 cm^2^, respectively, counting the points on the choroidal plaque and, if present, on the extra-chorionic area and lesions [22]. The necessary points were counted to obtain an error coefficient lower than 3.0% (Kappa test).

The reference values for the mean placental diameter according to the GA come from the study of Boyd and Hamilton [21]. Mean placental thickness was calculated from the placental area [22] and volume estimated by placental weight [24]. The fetal/placental coefficient obtained from the birthweight divided by the placental weight was also calculated [5,25]. The placental weight, area and thickness, birthweight, and fetal/placenta coefficients were transformed into a Z/GA score using the above criteria and by comparing with reference values contained in the literature [21,22,24,26]. The cut-off points chosen for the Z score were -1.28 < Z < +1.28, from which three growth categories were defined: small for gestational age (SGA)–Z / gestational age (GA) ≤ -1.28, suitable for gestational age (AGA)—Z / GA between -1.28 and +1.28, and large for gestational age (LGA): Z / GA ≥ +1.28 [27].

The hospital manager authorized the study, and the data collected remained confidential. The Research Ethics Committee of the Federal University of Espírito Santo approved this study under number 2,518,900 / 2018.

## Results

We identified 187 requests for fetal annexes examination at the university hospital during the study period. 65 cases (35%) were excluded because of technical problems with the specimens, and the final sample consisted of 122 cases (65.2%). A comparison of cases included and excluded from the series, showed that there were no disparities between variables like maternal age, ethnicity, education, parity, immunological status, and fetal morphology.

The mean age of women was 28.6 years with a standard deviation of (SD) = + 5.7 (22.9–34), 81.9% of the women had at least one previous gestation (100/122), 66.4% had six or more prenatal consultations (81/122), 33.0% were underweight or obese (33/100), 66.4% were diagnosed before the current gestation (81/122), 55.7% had AIDS criteria (68/122), and 52.4% had detectable viral load (64/122). BMI was associated with placental weight (p 0.024; see Table 1).

**Table 1:** Demographic and clinical variables, obstetric data and delivery distribution by placental weight and area in HIV-infected pregnant women.

Variable	Placental Weight		p value**	Placental Area		p value**
	SGA n (%)	Not SGA n (%)		SGA n (%)	Not SGA n (%)	
**Age (years)**						
17–35	23 (21.7)	83 (78.3)	0.521	34 (32.1)	72 (67.9)	0.400
36 or older	2 (12.5)	14 (87.5)		7 (43.7)	9 (56.3)	
**Ethnicity**						
White	5 (16.1)	26 (83.9)	0.610	8 (25.8)	23 (74.2)	0.380
Other	20 (22.0)	71 (78.0)		33 (36.3)	58 (63.7)	
**Education**						
< = 4 years	7 (20,0)	28 (80.0)	0.999	12 (34.3)	23 (65.7)	0.999
>4 years	18 (20.7)	69 (79.3)		29 (33.3)	58 (66.7)	
**Parity**						
Primigravida	7 (31.8)	15 (68.2)	0.154	33 (33.0)	67 (67.0)	0.806
More than one	18 (18.0)	82 (82.0)		8 (36.4)	14 (63.6)	
**Antenatal care**						
Less than six visits	17 (21.0)	64 (79.0)	0.388	11 (26.8)	30 (73.2)	0.313
Six or more visits	8 (19.5)	33 (80.5)		30 (37.0)	51 (63.0)	
**Tobacco use**						
No	20 (21.7)	72 (78.3)	0.614	32 (34.8)	60 (65.2)	0.664
Yes	5 (16.7)	25 (83.3)		9 (30.0)	21 (70.0)	
**Alcohol**						
No	18 (20.0)	72 (80,0)	0.999	33 (36.7)	57 (63.3)	0.280
Yes	7 (21.9)	25 (78.1)		8 (25.0)	24 (75.0)	
**Drug abuse**						
No	25 (22.3)	87 (77,7)	0.212	39 (34.8)	73 (65.2)	0.493
Yes	0 (0.0)	10 (100.0)		2 (20.0)	8 (80.0)	
Preeclampsia*						
No	22 (22.0)	78 (78.0)	0.454	31 (31.0)	69 (69.0)	0.518
Yes	1 (8.3)	11 (91.7)		5 (41.7)	7 (58.3)	
**Gestational diabetes**						
No	25 (21.2)	93 (78.8)	0.713	39 (33.0)	79 (67.0)	0.333
Yes	0 (0.0)	4 (100.0)		2 (50.0)	2 (50.0)	
**BMI** (kg/m^2^)*						
Underweight	6 (50.0)	6 (50.0)		4 (33.3)	8 (66.6)	
Normal weight	18 (24.0)	57 (76.0)	0.024	28 (37.3)	47 (62.6)	0.262
Overweight	1 (4.0)	24 (96.0)		9 (36.0)	16 (64.0)	
Obese	0 (0.0)	8 (100.0)		0 (0.0)	8 (100.0)	
**Urinary infection**						
No	23 (23.0)	77 (77.0)	0.241	35 (35.0)	65 (65.0)	0.620
Yes	2 (9.1)	20 (90.9)		6 (27.3)	16 (72.7)	
**Syphilis**						
No	24 (21.1)	90 (78,9)	0.999	40 (35.1)	74 (64.9)	0.265
Yes	1 (12.5)	7 (87.5)		1 (12.5)	7 (87.5)	
**Delivery**						
Vaginal	5 (23.8)	16 (76.2)	0.767	10 (47.6)	11 (52.4)	0.203
C-section	20 (19.8)	81 (80.2)		31 (30.7)	70 (69.3)	

*Missing values

**Fisher exact or chi square test; BMI: Body Mass Index

SGA placental weights were observed in 25 of 122 cases (20.5%), AGA in 96 (78.7%), and LGA in one case (0.8%). The mean placental weight was 364.5 ± 88.3 g, ranging from 84 to 569 g and the Z/GA score of placental weight ranged from -3.4 to 1.9. It was possible to evaluate placental thickness in all cases, varying between 8.0 and 28.0 mm, with a mean of 18 ± 3.4 mm. The thickness Z/GA score ranged from -2.7 to 3.3. SGA thickness occurred in 4.9% of cases (6/122), a lower occurrence than the reference value of 10% corresponding to a DI of -5.1%.

Measurement of the placental area identified a variation between 107 and 290 cm^2^, with an average of 205.7 ± 41.3 cm^2^. The Z/GA score of placental area ranged from -2.7 to 0.8. SGA placental area was identified in 33.6% of the cases (41/122), There was no occurrence of GIG placental area.

The median T-CD4 + lymphocyte count in the pregnant women was 441 mm^3^, with interquartile range (IQR) = 303–620. Counts below 350 cells per mm^3^ of blood were detected in 42 cases and below 200 mm^3^ in 16. The median viral load (VL) was 601 copies/ml (IQR) = 1–6,765, of the women with detectable viral load, and the median was 2,027.5 (IQR = 618.5–26,849). VL was observed at or above 1,000 copies/ml in 47.5% (58/122) and undetectable in 34.4% (42/122) of the cases. Regarding ART received during pregnancy, 86.8% of the pregnant women (106/122) received some antiretroviral therapy, 8.4% received Zidovudine monotherapy (9/106), 1.8% received dual therapy (2/106), and 89.6% (95/106) received high-potency ART. Of these, 89.5% (85/95) received ART with PI and 10.5% (10/95) without PI. Among the 16 pregnant women who did not receive ART during prenatal care (13.1%), three did not receive intravenous ZDV during childbirth (18.7%; see Table 2). Of the 16 women who did not receive ART, four did not receive prenatal care, and eight were diagnosed with HIV infection at the end of pregnancy.

**Table 2:** HIV infection characteristics in pregnant women and placenta dimensions distribution by placental weight and area.

Variable	Placental Weight		p value*	Placental Area		p value*
	SGA n (%)	Not SGA n (%)		SGA n (%)	Not SGA n (%)	
**HIV diagnosis**						
Before pregnancy	17 (21.0)	64 (79.0)	0.999	27 (33.3)	54 (66.7)	0.999
During pregnancy	8 (19.5)	33 (80.5)		14 (34.1)	27 (65.9)	
**HIV status**						
HIV	11 (20.8)	42 (79.2)	0.822	17 (32.1)	36 (67.9)	0.847
AIDS	14 (20.3)	55 (79.7)		24 (34.8)	45 (65.2)	
**CD4 counts**						
< 200 copies	5(31.3)	11(68.8)	0.450	6 (37.5)	10 (62.5)	
200 to 499 copies	9 (16.7)	45 (83.3)		13 (24.1)	41 (75.9)	0.999
> = 500 copies	11(21.2)	41(78.8)		22 (42.3)	30 (57.7)	
**CD4 counts**						
< = 350 copies	10 (23.8)	32 (76.2)	0.637	14 (33.3)	28 (66.7)	0.086
> 350 copies	15 (18.8)	65 (81.3)		27 (33.7)	53 (66.3)	
**Viral load**						
Undetectable	9 (21.4)	33 (78.6)	0.999	16 (38.1)	26 (61.9)	0.546
Detectable	16 (20.0)	64 (80.0)		25 (31.2)	55 (68.8)	
**ART initiation**						
Without ART	4 (25.0)	12 (75.0)	0.437	3 (30.0)	7 (70.0)	0.999
Since before pregnancy	6 (16.2)	31 (83.8)		13 (34.2)	25 (65.8)	
In pregnancy	15 (21.7)	54 (78.3)		25 (33.8)	49 (66.2)	
**Protease inhibitor**						
No	9 (25.0)	27 (75.0)	0.798	15 (48.4)	16 (51.6)	**0.049**
Yes	16 (18.6)	70 (81.4)		26 (28.6)	65 (71.4)	
**Placental thickness**						
SGA	5 (83.3)	1 (16.7)	**< 0.001**	2 (33.3)	4 (66.7)	
AGA	20 (20.6)	77 (79.4)		30 (30.9)	67 (69.1)	**0.383**
LGA	0 (0.0)	19 (100.0)		9 (47.4)	10 (52.6)	
**Placental Area**						
SGA	19 (46.3)	22 (53.7)	**< 0.001**	-	-	-
AGA	6 (7.4)	75 (92.6)		-	-	-
**Placental weight**						
SGA	-	-	-	6 (24.0)	19 (76.0)	
AGA	-	-	-	74 (77.1)	22 (22.9)	**< 0.001**
LGA	-	-	-	1 (100.0)	0 (0.0)	

*Fisher exact or chi square test; ART–antiretroviral therapy; SGA–small for gestational age; AGA—adequate for gestational age; LGA—large for gestational age.

Regarding placenta data, it was observed that SGA placental thickness was associated to SGA placental weight (p<0.001) but was not associated to placental area (p.0383). SGA placental area was associated to SGA placental weight (p<0.001).

Table 3 describes the fetal data, the outcome of the child, and mother-to-child-transmission (MTCT). Apgar scores in the first minute of seven or more were observed in 113 of 122 cases (92%). There was HIV vertical transmission in 6 of 122 (4.9%), in four of them there was a SGA placenta area (4/6, 66.7%), and in one case (1/6, 16.7%) there was a simultaneous occurrence of SGA for birth weight, placental weight, and placental area. The gestational outcome observed in this study included 15.6% (19/122) preterm births and 84.4% births at term (103/122). Regarding the classification of GA in the termination according to maternal immunological status, 73.7% of the cases of preterm birth in children of pregnant women with AIDS criteria were observed (14/19). Among the six cases of indicated preterm delivery observed in this series, three occurred because of severe pre-eclampsia, one was associated with maternal coma, one had a zero diastole on fetal ultrasonography, and another had a placenta previa with fetal death. AIDS criteria was present in five cases of iatrogenic preterm. Admission in neonatal intensive care unit occurred in 22.1% of the cases (27/122), and of these, 51.8% were preterm. AGA birthweight was associated with a non SGA placental weight (85.9%) compared to SGA birthweight (14.1%). AGA birthweight was associated with a non SGA placental area (70.7%) compared to SGA birthweight (29.3%).

**Table 3:** Fetal variables, perinatal outcomes and MTCT in HIV-infected pregnant women distribution by weight and placental area.

Variable	Placental Weight		p value*	Placental Area		p value*
	SGA n (%)	Not SGA n (%)		SGA n (%)	Not SGA n (%)	
**Fetal gender**						
Male	9 (15.8)	48 (84.2)	0.266	19 (33.3)	38 (66.7)	0.999
Female	16 (24.6)	49 (75.4)		22 (33.8)	43 (66.2)	
**Apgar score at first minute**						
< 7	2 (28.6)	5 (71.4)	0.426	4 (44.4)	5 (55.6)	0.483
≥ 7	22 (19.5)	91 (80.5)		37 (32.7)	76 (67.3)	
**Preterm birth**						
Up to 36 weeks	6 (31.6)	13 (68.4)	0.219	8 (42.1)	11 (57.9)	0.433
37 or more weeks	19 (18.4)	84 (81.6)		33 (32.0)	70 (68.0)	
**Perinatal death**						
Yes	2 (40.0)	3 (60.0)	0.271	40 (34.2)	77 (65.8)	0.662
No	23 (19.7)	94 (80.3)		1 (20.0)	4 (80.0)	
**Weight at birth**						
< 2,500 g	13 (48.1)	14 (51.9)	**<0.001**	14 (51.9)	13 (48.1)	**0.036**
≥ 2,500 g	12 (12.6)	83 (87.4)		27 (28.4)	68 (71.6)	
**Weight at birth/GA**						
SGA	12 (50.0)	12 (50.0)	**0.001**	14 (58.3)	10 (41.7)	
AGA	13 (14.1)	79 (85.9)		27 (29.3)	65 (70.7)	**0.006**
LGA	0 (0.0)	6 (100.0)		0 (0.0)	6 (100.0)	
**MTCT of HIV**						
No	24 (20.7)	92 (79.3)	0.999	37 (31.9)	79 (68.1)	0.097
Yes	1 (16.7)	5 (83.3)		4 (66.7)	2 (33.3)	

*Fisher exact or chi square test; MTCT–mother-to-child-transmission; SGA–small for gestational age; AGA—adequate for gestational age; LGA—large for gestational age.

Death was the observed result in 4.1% of the pregnancies (5/122), fetal death occurred in two cases and neonatal in three. There was one case of septicemia in the preterm condition, one case of fetus at term, whose death occurred in another institution and could not be evaluated, and one case of chorioamnionitis due to premature rupture of membranes in a preterm fetus. Weight at birth > = 2,500gr and AGA weight at birth/GA were associated to not SGA placental weight and area; see Table 3.

In the multivariate analysis of the association between preterm birth, a 74% reduction in the probability of preterm birth was observed in women who attended six or more prenatal visits (p = 0.046), and 93% of the newborns had Apgar scores equal to or above seven (p = 0.004). For women 36 years of age or older, they were 5.7 times more likely to have a preterm birth than those under 36 (p = 0.044). For patients with AIDS-defining criteria, they were 3.7 times more likely to have preterm births (see Table 4). The birth weight was evaluated in all cases, ranging from 374 to 5,015g with a mean of 2,821.1 ± 678.2g. The Z/GA score of birthweight ranged from -6.2 to 5.3. SGA birthweight was verified in 19.7% of the cases (24/122), higher than the expected occurrence of 10% for the general population corresponding to a direct increase (DI) of 9.7%.

**Table 4:** Association between preterm birth and variables that remained in the final multivariate analysis.

Outcome	Independent Variables		p value*	OR adjusted (CI 95%)
**Preterm birth**	Maternal status	HIV	-	1
		AIDS	0.131	3.66 (0.68–19.67)
	Antenatal care	< 6 visits	-	1
		≥ 6 visits	**0.046**	0.26 (0.07–0.98)
	Anemia	No	-	1
		Yes	0.790	1.24 (0.26–5.85)
	Apgar score first minute	< 7	-	1
		≥ 7	**0.004**	0.07 (0.01–0.42)
	Age	< 36 years old	-	1
		≥ 36 years old	**0.044**	5.66 (1.05–30.62)
	ART initiation	Without ART	-	1
		Since before pregnancy	0.290	0.30 (0.03–2.80)
		During pregnancy	0.120	0.21 (0.03–1.50)
**Spontaneous preterm birth**	HAART	No**	-	1
		Without ART	0.999	0.00 (0.00)
		Yes	0.739	2.56 (0.01–64.69)
	Antenatal care	< 6 visits	-	1
		≥ 6 visits	0.066	0.21 (0.04–1.11)
	Anemia	No	-	1
		Yes	0.774	1.31 (0.20–8.43)
	Apgar score first minute	< 7	-	1
		≥ 7	**0.004**	0.04 (0.01–0.37)
	Age	< 36 years old	-	1
		≥ 36 years old	0.098	5.87 (0.72–47.55)
	ART initiation	Without ART	-	1
		Since before pregnancy	0.999	0.00 (0.00)
		During pregnancy	0.999	0.00 (0.00)
**Indicated preterm birth**	Age	< 36 years old	-	1
		≥ 36 years old	0.098	4.65 (0.75–28.65)

* Multivariate analysis

**One or two antiretroviral drugs; ART antiretroviral therapy; HAART—Highly active antiretroviral therapy; OR—*Odds Ratio*; (1) Reference

The variables of weight, area, and placental thickness were input into a multivariate analysis along with variables that remained in the model, with statistically significant differences between SGA placental weight and SGA placental thickness (p = 0.003), and between placental weight and placental area (p = 0.001; Table 5). An association was observed between AGA birthweight and HAART usage (p = 0.030). Of 92 AGA fetuses, 75 mothers (81.5%) used HAART, six received mono or dual therapy (6.5%), and 11 received no ARV (12.0%). Regarding SGA newborns, 17 used HAART (70.8%), 5 used mono or dual therapy (20.8%), and two did not take ARV (8.3%). Among LGA fetuses, three received HAART (50.0%), and three did not take any ARV (50.0%). A statistically significant association was observed in the multivariate analysis between the AGA placental area and the use of PI (p = 0.047, OR = 0.34 [0.12–0.98]), and between the SGA placental weight and SGA area (p = <0.001, OR = 0.11 [0.04–0.35]).

**Table 5:** Association between placental weight and area with variables that remained in the final multivariate analysis.

**Variable**	**Placental Weight**		**OR Crude (CI 95%)**	p value*	**OR Adjusted (CI 95%)**	p value**
	**SGA n (%)**	**Not SGA n (%)**				
**P. Thickness**						
SGA	5 (83.3)	1 (16.7)	1		1	
AGA	20 (20.6)	77 (79.4)	0.05 (0.01–0.47)	0.008	0.02 (0.00–026)	**0.003**
LGA	0 (0.0)	19 (100.0)	0.00 (0.00-)	0.998	0.00 (0.00-)	0.998
**Placental Area**						
SGA	19 (46.3)	22 (53.7)	1		1	
AGA	6 (7.4)	75 (92.6)	0.09 (0.03–0.26)	**< 0.001**	0.05 (0.01–0.21)	**< 0.001**
**Weight at birth**						
<2,500 g	13 (48.1)	14 (51.9)	1		1	
≥2,500 g	12 (12.6)	83 (87.4)	0.16 (0.06–0.41)	**< 0.001**	0.43 (0.11–1.68)	0.224
**Weight at birth/GA**						
SGA	12 (50.0)	12 (50.0)	1		1	
AGA	13 (14.1)	79 (85.9)	0.17 (0.06–0.44)	**< 0.001**	0.47 (0.12–1.88)	0.283
LGA	0 (0.0)	6 (100.0)	0.00 (0.00-)	0.999	0.00 (0.00)	0.999
**Variable**	**Placental Area**		**OR Crude (CI 95%)**	p value*	**OR Adjusted (CI 95%)**	p value**
	**SGA n (%)**	**Not SGA n (%)**				
**PI use**						
No	15 (48.4)	16 (51.6)	1		1	
Yes	26 (28.6)	65 (71.4)	0.43 (0.18–0.99)	**0.046**	0.34 (0.12–0.98)	**0.047**
**Placental weight**						
SGA	19 (76.0)	6 (24.0)	1		1	
AGA	22 (22.9)	74 (77.1)	0.09 (0.03–0.26)	**< 0.001**	0.11 (0.04–0.35)	**< 0.001**
LGA	0 (0.0)	1 (100.0)	0 (0.00-)	0.999	0 (0.00-)	0.999
**Weight at birth**						
<2,500 gr	14 (51.9)	13 (48.1)	1		1	
> = 2,500 gr	27 (28.4)	68 (71.6)	0.37 (0.15–0.89)	**0.026**	0.72 (0.21–2.51)	0.605
**Weight at birth/GA**						
SGA	14 (58.3)	10 (41.7)	1		1	
AGA	27 (29.3)	65 (70.7)	0.30 (0.12–0.75)	**0.010**	0.65 (0.19–2.22)	0.497
LGA	0 (0.0)	6 (100.0)	0 (0.00-)	0.999	0 (0.00-)	0.999

*Fisher exact or chi square test

** multivariate analysis; OR—Odds Ratio; GA gestational age; P. = Placental; SGA–small for gestational age; AGA—adequate for gestational age; LGA—large for gestational age.

## Discussion

The occurrence of 20.5% of placentas with SGA weight discovered in the present study is higher than established reference values [21,27], but it was similar to the 20% (7/35) verified in a case study conducted in the city of Niterói, Brazil [28]. In addition, it was less frequent in comparison with the 54.3% of SGA-weighted placentas (64/118) reported in pregnant women in Cape Town (South Africa) who had used predominantly HAART [29]. The mean value of placental weight was lower than the value reported in other cases of HIV-infected pregnant women [28,29,30]. Other data also found the highest placental mean weight among HIV-infected when compared to uninfected women [31,32]. In the present study, almost 80% of pregnant women used HAART, and there was an association between SGA placentas and low birth weight in contrast to the study by Kalk et al. that observed a weak correlation with weight at birth despite a high report rate of placentas with SGA weight [29].

Placental area serves as a marker of poor reproductive outcomes, of conditions and possible diseases in adult life, and it is a determinant of independent fetal growth [33]. In this sense, the occurrence of 33.6% of SGA placental area may indicate that some aggression or failure of adaptation occurred early in the pregnancy since the area reflects lateral growth of the placenta that is predominantly performed in the first and second gestational trimesters [5]. In addition, the association between the use of PI schemes and the occurrence of SGA placental area in this series may suggest an early effect of PI on lateral placental growth. However, there was no statistical association with maternal immunological status or comorbidities not associated with HIV, nor were there other studies that analyzed this dimension in pregnant women using PI regimens. The Z/GA score of the placental area was lower than previously referenced values reported in the literature about the human placenta [21,34].

Data from the National Collaborative Perinatal Project indicate that the presence of comorbidity may trigger a reflex of maladaptation or a compensatory mechanism that influences both reduction and placental hypertrophy. However, no studies on placenta size were identified in HIV-infected pregnant women that included an analysis of the placental area [4]. In this sense, and contrary to the other placental dimensions evaluated in this study, the high proportion of LGA placental thickness can be related to possible adaptive growth. An average placental thickness similar to the 20 mm reported in a sample from Niterói [28] was found, contrasting with results observed in a Nairobi study, with placentas significantly less thick at preterm terminations of HIV infected pregnant women when compared to women not infected with preterm deliveries [35]. The proportion of SGA weight was higher than that observed in other studies with HIV-infected pregnant women performed in São Paulo [36], in South Africa [9], and in the general population in Brazil [19]. There was also an occurrence of SGA fetal weight in 33.3% of the cases of pregnant women using illicit drugs, higher than the 25% reported in a similar group in São Paulo [36].

Regarding maternal immunological status, our data do not corroborate the hypothesis of more frequent SGA weight in cases of AIDS [37,38], with a high viral load and immunosuppression [38,39,40], that would represent an inability of the maternal organism to provide a supply appropriate to the fetus. When assessing the use of HAART, we identified higher proportion of AGA birthweight among women receiving HAART. This result differs from Chen et al. (2012) who found an association between SGA birthweight and the use of HAART [41]. Despite the high proportion of SGA born fetuses in this study and the presence of PI categorized ART, there was no statistical difference. These data differ from findings of a study that identified a higher risk for restricted fetal growth in pregnant women using regimens based on PI initiated before gestation [13], and from a study conducted by Van der Merwe et al. (2011) that showed an association between SGA birthweight and the use of PI regimens [42].

In the multivariate analysis of placental and birthweight in the present study, there was no significant difference in the relationship between SGA placental weight and SGA birthweight. Among the cases of SGA birthweigh, there was higher occurrence of SGA placental weight and SGA placental area. These findings may indicate that SGA placental weight and area precede fetal growth disorders and can therefore serve as a predictor, thus substantiating the proposed measurement of placental area and volume in the second trimester as markers of possible early aggression as well as predictors of perinatal morbidity. An important implication is that placental growth disorders are the most sensitive markers of aggression. In contrast, a restriction of placental growth may not be concomitant with the restriction of fetal growth, either because the fetus has optimized its growth or because delivery was anticipated [43].

Sociodemographic profile of HIV-infected pregnant women in this study reveals vulnerabilities that allow them to be characterized as an “at-risk group” for adverse reproductive outcomes, similar to that observed in both local [44,45] and Brazilian cases [36,46]. In this context, it is imperative not only to recognize risk factors like maternal age and BMI disorders [4] and conduct the corresponding interventions but also to promote comprehensive care in all circumstances for these women, infected or not, by strictly controlling growth [15,47]. Maternal nutritional status is associated with better placental growth, including higher periconceptional BMI and gestational weight gain [4].

The high occurrence of cesarean sections observed in this group is as a result of following the Ministry of Health recommendations that elective cesarean sections should be conducted for HIV-infected pregnant women before the beginning of labor and the rupture of membranes to reduce vertical transmission rate. Also, maternal plasma viral load is the most substantial predictive factor of fetal infection risk, and detectable viral load occurred in more than 60% of the cases, thus possibly justifying the obstetric decision of non-vaginal delivery [18].

The proportion of preterm birth was higher than that reported by Lawn [48] for developed countries and by a study performed in Rio de Janeiro [49]; however, it was similar to the prevalence of HIV-infected pregnant women in Europe [50], South Africa [9,42], and in another Brazilian study [51]. It was,however, lower in comparison with the study in São Paulo [52]. The following factors were associated with preterm birth: Apgar scores below seven, women with less than six prenatal consultations, and maternal ages above 36 years. The probability of preterm birth was 3.7 times higher in pregnant women who had AIDS-defining criteria. A maternal age equal to or above 36 years was a risk factor that increased the probability of preterm birth by 5.7 times, spontaneous preterm birth by 5.9 times, and indicated preterm birth by 4.6 times compared to pregnant women under 36 years of age. Wang et al. found no association between maternal age and preterm birth [53].

In our study, pregnant women using HAART had a 2.6-fold higher probability of spontaneous preterm birth than those who did not. In this group, there was no association between PI use and preterm birth, possibly due to the significant number of women who used antiretroviral therapy with PI during gestation. Watts et al. found an association between the use of protease inhibitor-initiated regimens before gestation and the increased risk of preterm birth [54]. These results were also evidenced in other studies [49,55]. However, not all studies adjusted the maternal immune status that, by itself, could increase the risk of prematurity.

Regarding vertical transmission, it was observed that all cases occurred with some associated risk factors like syphilis, lack of prenatal care and no antiretroviral use. The 4.9% MTCT rate was higher than the 2.6% prevalence rate reported for Brazil [52].

Regarding fetal deaths, two cases occurred in preterm cases before 32 weeks in the present study. A high risk of fetal death has been reported in HIV-infected pregnant women, particularly in developing countries [56]. Some factors of morbidity besides virus infection, such as the use of illicit drugs or unfavorable socioeconomic conditions, are described as being possibly responsible for the high risk of fetal death observed in these populations. The 4.1% prevalence of perinatal death found in the study was above the SINASC values [19].

It should be highlighted that one limitation of this study may have been the selection of cases from a reference hospital that treats more cases of high obstetric risks, thereby explaining the higher prevalence of AIDS cases, medical complications, gestational complications, and poor reproductive outcomes compared to other health services. The proportion of cases with AIDS-defining criteria was higher than that reported in a previous study conducted in the same region [44]. However, there was no statistically significant difference when comparing maternal immune status and placental weight by GA. Other limitations of this study are the long period over which it was conducted, a decision adopted to increase the number of cases, as well as the lack of a comparative group of non-HIV infected pregnant women as is recommended by some authors [29,57]. However, the present study used the reference values for placental dimensions and fetal weight already contained in the literature [58].

## Conclusions

The findings of the present study point out higher prevalence of placental growth disorders in HIV-infected pregnant women compared to international reference values. This may be due to the effects of the virus or to the use of combined antiretroviral regimens. The restriction of placental growth was the most common disorder, as evidenced by the high occurrence of SGA placental weight and SGA placental area, which are statistically related to the use of antiretroviral regimens with protease inhibitors. There was also an LGA thickness frequency above that expected in this study, probably the result of a compensatory mechanism to the other disorders. Regarding adverse reproductive outcomes, preterm birth and perinatal death were the most observed outcomes in HIV-infected pregnant women in this casuistry, and they occurred more frequently than the reference values specified. We suggest that multicentric studies with a more significant number of cases should be performed to increase knowledge about placental growth disorders and adverse reproductive outcomes in HIV-infected pregnant women, thereby contributing to the identification and prevention of morbidity and mortality factors in this group.

## Supporting information

S1 Data (SAV)

## References

[1] Fox H, Elston CW. Pathology of the placenta. Major Probl Pathol. 1978;7: 1–491.4 207935

[2] Vargas PRM. Placental examination: A challenge for pathologists. J. Bras. Patol. Med. Lab. [Internet]. 2013 12 [cited 2018 July 01]; 49(6): 386–387.

[3] Ismail KI, Hannigan A, Kelehan P, Fitzgerald B, O'Donoghue K, Cotter A. Small for gestational age infants and the association with placental and umbilical cord morphometry: A digital imaging study. J Matern Fetal Neonatal Med. 2019 2 27: 1–8. 10.1080/14767058.2019.1582628 30760075

[4] Baptiste-Roberts K, Salafia CM, Nicholson WK, Duggan A, Wang NY, Brancati FL. Maternal risk factors for abnormal placental growth: The national collaborative perinatal project. BMC Pregnancy Childbirth. 2008 9 23;8: 44 10.1186/1471-2393-8-44 18811957 PMC2564930

[5] Salafia CM, Charles AK, Maas EM. Placenta and fetal growth restriction. Clin Obstet Gynecol. 2006 6;49(2): 236–56. 10.1097/00003081-200606000-00007 16721104

[6] Yampolsky M, Salafia CM, Shlakhter O, Haas D, Eucker B, Thorp J. Modeling the variability of shapes of a human placenta. Placenta. 2008 9;29(9): 790–7. 10.1016/j.placenta.2008.06.005 18674815 PMC2570048

[7] Egbor M, Ansari T, Morris N, Green CJ, Sibbons PD. Morphometric placental villous and vascular abnormalities in early- and late-onset pre-eclampsia with and without fetal growth restriction. BJOG. 2006 5;113(5): 580–9. 10.1111/j.1471-0528.2006.00882.x 16579806

[8] Salavati N, Sovio U, Mayo RP, Charnock-Jones DS, Smith GC. The relationship between human placental morphometry and ultrasonic measurements of utero-placental blood flow and fetal growth. Placenta. 2016 2;38: 41–8. 10.1016/j.placenta.2015.12.003 26907381

[9] Ndirangu J, Newell ML, Bland RM, Thorne C. Maternal HIV infection associated with small-for-gestational age infants but not preterm births: Evidence from rural South Africa. Hum Reprod. 2012;27: 1846–56. 10.1093/humrep/des090 22442245 PMC3357196

[10] Li N, Sando MM, Spiegelman D, Hertzmark E, Liu E, Sando D, et al Antiretroviral Therapy in Relation to Birth Outcomes among HIV-infected Women: A Cohort Study. J Infect Dis. 2016 Apri 1;213(7): 1057–64. 10.1093/infdis/jiv389 26265780

[11] Dos Reis HL, Araujo Kda S, Ribeiro LP, Da Rocha DR, Rosato DP, Passos MR, et al Preterm birth and fetal growth restriction in HIV-infected Brazilian pregnant women. Rev Inst Med Trop Sao Paulo. 2015 Mar-Apr;57(2): 111–20 10.1590/S0036-46652015000200003 25923889 PMC4435008

[12] Chetty T, Thorne C, Coutsoudis A. Preterm delivery and small-for-gestation outcomes in HIV-infected pregnant women on antiretroviral therapy in rural South Africa: Results from a cohort study, 2010–2015. PLoS One. 2018 2 22;13(2). 10.1371/journal.pone.0192805 PMC5823389 29470508

[13] Snijdewind IJM, Smit C, Godfried MH, et al Preconception use of cART by HIV-positive pregnant women increases the risk of infants being born small for gestational age. PLoS One. 2018;13(1):e0191389 10.1371/journal.pone.0191389 29351561 PMC5774764

[14] Uthman OA, Nachega JB, Anderson J, Kanters S, Mills EJ, Renaud F, et al Timing of initiation of antiretroviral therapy and adverse pregnancy outcomes: A systematic review and meta-analysis. Lancet HIV. 2017 1;4(1):e21–e30. 10.1016/S2352-3018(16)30195-3 27864000

[15] Fausto MA, CarneiroII M, Antunes CMF, Colosimo EA, Pinto JA. Avaliação antropométrica longitudinal de lactentes nascidos de mães infectadas pelo HIV [Longitudinal anthropometric assessment of infants born to HIV-infected mothers]. Rev Saúde Pública 2011;45(4): 652–60. 10.1590/s0034-89102011005000040 21670861

[16] Blair JM, Hanson DL, Jones JL, Dworkin MS. Trends in pregnancy rates among women with human immunodeficiency virus. Obstet Gynecol. 2004 4;103(4): 663–8. 10.1097/01.AOG.0000117083.33239.b5 15051556

[17] Gardosi J, Mongelli M, Wilcox M, Chang A. An adjustable fetal weight standard. Ultrasound Obstet Gynecol 1995; 6: 168–74. 10.1046/j.1469-0705.1995.06030168.x 8521065

[18] Brasil. Ministério da Saúde. Secretaria de Vigilância em Saúde. Departamento de Vigilância Epidemiológica. Guia de vigilância em saúde [Ministry of Health. Secretariat of Health Surveillance. Department of Epidemiological Surveillance. Health surveillance guide]. 3a ed. Brasília: Ministério da Saúde; 2019.

[19] Brasil. Ministério da Saúde. Secretaria de Vigilância em Saúde. Informações de Saúde (TABNET). Estatísticas Vitais. Nascidos Vivos—1994 a 2016 [Ministry of Health. Health Surveillance Secretariat. Health Information (TABNET). Vital statistics. Live Born—1994 to 2016]. Brasília: MS/SVS; 2016. Accessed: October 20th, 2018. Available at http://www2.datasus.gov.br/DATASUS/index.php?area=0205&id=6936&VObj=http://tabnet.datasus.gov.br/cgi/deftohtm.exe?sinasc/cnv/nv

[20] Goldenberg RL, Culhane JF, Iams JD, Romero R. Epidemiology and causes of preterm birth. Lancet. 2008 1 5;371(9606): 75–84. 10.1016/S0140-6736(08)60074-4 18177778 PMC7134569

[21] Boyd JD, Hamilton WJ. The Human Placenta. Cambridge: W. Heffer and Sons Ltd; 1970.

[22] Weibel ER. Stereological methods: Theoretical foundations. Academic Press; 1980.

[23] Howard CV, Reed M. Unbiased Stereology three-dimensional measurement in microscopy, second edition, Garland Science/BIOS Scientific Publisher; 2005.

[24] Hellman LM, Kobayashi M, Tolles WE, Cromb E. Ultrasonic studies on the volumetric growth of the human placenta. Am J Obstet Gynecol. 1970 11 1;108(5): 740–50. 10.1016/0002-9378(70)90540-5 5471239

[25] Molteni RA, Stys SJ, Battaglia FC. Relationship of fetal and placental weight in human beings: Fetal/placental weight ratios at various gestational ages and birth weight distributions. J Reprod Med. 1978 11;21(5): 327–34. 731626

[26] Salafia CM, Yampolsky M. Metabolic scaling law for fetus and placenta. Placenta. 2009 5;30(5): 468–71. 10.1016/j.placenta.2008.12.013 19285342 PMC2699210

[27] Feinstein AR. Principles of medical statistics. London: Chapman Hall; 2002.

[28] López CL, Pires ARC, Fonseca EC, Rodrigues FR, Braga Neto AR, Herdy GVH. Anatomopathological characterization of placentas from HIV+ patients associated with p24 expression. J. Bras. Patol. Med. Lab. 2013 12; 49(6): 437–445.

[29] Kalk E, Schubert P, Bettinger JA, Cotton MF, Esser M, Slogrove A, et al Placental pathology in HIV infection at term: A comparison with HIV-uninfected women. Trop Med Int Health. 2017 5;22(5): 604–613. 10.1111/tmi.12858 28214384

[30] Schwartz DA, Sungkarat S, Shaffer N, Laosakkitiboran J, Supapol W, Charoenpanich P, et al Placental abnormalities associated with human immunodeficiency virus type 1 infection and perinatal transmission in Bangkok, Thailand. J Infect Dis. 2000 12;182(6): 1652–7. 10.1086/317634 11069236

[31] Jauniaux E, Nessmann C, Imbert MC, Meuris S, Puissant F, Hustin J. Morphological aspects of the placenta in HIV pregnancies. Placenta. 1988 Nov-Dec;9(6): 633–42. Erratum in: Placenta 1989 May-Jun;10(3): 320. 10.1016/0143-4004(88)90007-0 3257096

[32] Gichangi PB, Nyongo AO, Temmerman M. Pregnancy outcome and placental weights: Their relationship to HIV-1 infection. East Afr Med J. 1993 2;70(2): 85–9. 8513748

[33] Barker DJ, Thornburg KL, Osmond C, Kajantie E, Eriksson JG. The surface area of the placenta and hypertension in the offspring in later life. Int J Biol. 2010;54(2–3): 525–30. 10.1387/ijdb.082760db PMC3923649 19876839

[34] Benirschke K, Kaufmann P & Baergen R. The pathology of the human placenta. 5 ed. New York: Springer-Verlag; 2006.

[35] Obimbo MM, Zhou Y, McMaster MT, Cohen CR, Qureshi Z, Ong'ech J, et al Placental Structure in Preterm Birth Among HIV-Positive Versus HIV-Negative Women in Kenya. J Acquir Immune Defic Syndr. 2019 1 1;80(1): 94–102. 10.1097/QAI.0000000000001871 30272633 PMC6289800

[36] Lopes MAB, Bunduki V, Ruocco RMSA, Lopes LM, Tavarez G, Zugaib M. Avaliação ultra-sonográfica, ecocardiográfica fetal e resultados perinatais em gestantes portadoras do HIV em uso de terapia anti-retroviral [Fetal ultrasound, echocardiographic evaluation and perinatal outcomes in HIV-positive pregnant women using antiretroviral therapy]. Rev. Bras. Ginecol. Obstet. 2007 10; 29(10): 497–505.

[37] Coley l.L., Msamanga G.I, Fawzi M.C., Kaaya S., Hertzmark E., Kapiga S., et al The association between maternal HIV-1 infection and pregnancy outcomes in Dar-es-Salaam, Tanzania. Br J Obstet Gynaecol. 2001;108(11): 1125–1133. 10.1111/j.1471-0528.2003.00269.x PMC6276363 11762650

[38] Aaron E, Bonacquisti A, Mathew L, Alleyne G, Bamford LP, Culhane JF. Small-for-gestational-age births in pregnant women with HIV, due to severity of HIV disease, not antiretroviral therapy. Infect Dis Obstet Gynecol. 2012:135030 10.1155/2012/135030 22778533 PMC3388287

[39] Iqbal SN, Kriebs J, Harman C, Alger L, Kopelman J, Turan O, et al Predictors of fetal growth in maternal HIV disease. Am JPerinatol. 2010 8;27(7): 517–23. 20200807 10.1055/s-0030-1248937

[40] Cailhol J, Jourdain G, Coeur SL, Traisathit P, Boonrod K, Prommas S, et al; Perinatal HIV Prevention Trial Group. Association of low CD4 cell count and intrauterine growth retardation in Thailand. J Acquir Immune Defic Syndr. 2009 4 1;50(4): 409–13. 10.1097/QAI.0b013e3181958560 19214117

[41] Chen JY, Ribaudo HJ, Souda S, et al Highly active antiretroviral therapy and adverse birth outcomes among HIV-infected women in Botswana. J Infect Dis 2012; 206: 1695–705. 10.1093/infdis/jis553 23066160 PMC3488194

[42] Van der Merwe K, Hoffman R, Black V, Chersich M, Coovadia A, Rees H. Birth outcomes in South African women receiving highly active antiretroviral therapy: A retrospective observational study. J Int AIDS Soc. 2011 8 15;14: 42 10.1186/1758-2652-14-42 21843356 PMC3163172

[43] Kalanda BF, van Buuren S, Verhoeff FH, Brabin BJ. Anthropometry of fetal growth in rural Malawi in relation to maternal malaria and HIV status. Arch Dis Child Fetal Neonatal Ed. 2005 3;90(2): F161–5. 10.1136/adc.2004.054650 15724042 PMC1721866

[44] Miranda AE, Soares RA, Prado BC, Monteiro RB, Figueiredo NC. Mother to child transmission of HIV in Vitória, Brazil: Factors associated with lack of HIV prevention. AIDS Care. 2005;17: 721–8. 10.1080/09540120500038033 16036258

[45] do Prado TN, Brickley DB, Hills NK, Zandonade E, Moreira-Silva SF, Miranda AE. Factors Associated with Maternal-Child Transmission of HIV-1 in Southeastern Brazil: A Retrospective Study. AIDS Behav. 2018 7;22(Suppl 1): 92–98. 10.1007/s10461-018-2172-8 29845389 PMC6045966

[46] Melo VH, Maia MMM, Correa Júnior MD, Kakehasi FM, Ferreira FGF, Andrade BAM, et al Vertical Transmission of HIV-1 in the Metropolitan Area of Belo Horizonte, Brazil: 2006–2014. Rev Bras Ginecol Obstet. 2018 2;40(2): 59–65. 10.1055/s-0037-1613689 29253912 PMC10309331

[47] Lartey A, Marquis GS, Mazur R, Perez-Escamilla R, Brakohiapa L, Ampofo W, et al Maternal HIV is associated with reduced growth in the first year of life among infants in the Eastern region of Ghana: The Research to Improve Infant Nutrition and Growth (RIING) Project. Matern Child Nutr. 2014 10;10(4): 604–16. 10.1111/j.1740-8709.2012.00441.x 22905700 PMC4193668

[48] Lawn JE, Cousens SN, Darmstadt GL, Bhutta ZA, Martines J, Paul V, et al; Lancet Neonatal Survival Series steering team. 1 year after The Lancet Neonatal Survival Series—was the call for action heard? Lancet. 2006 5 6;367(9521): 1541–7 10.1016/S0140-6736(06)68587-5 16679168 PMC7138031

[49] Machado ES, Hofer CB, Costa TT, et al Pregnancy outcome in women infected with HIV-1 receiving combination antiretroviral therapy before versus after conception. Sex Transm Infect. 2009; 85: 82–87. 10.1136/sti.2008.032300 18987014 PMC2864649

[50] Boer K, Nellen JF, Patel D, Timmermans S, Tempelman C, Wibaut M, et al The AmRo study: Pregnancy outcome in HIV-1-infected women under effective highly active antiretroviral therapy and a policy of vaginal delivery. BJOG. 2007 2;114(2): 148–55. 10.1111/j.1471-0528.2006.01183.x 17305888

[51] da Costa TP, Leal MC, Mota JC, Machado ES, Costa E, Vianna P, et al Comparison of pregnancy characteristics and outcomes between HIV-infected and HIV-non-infected women in Brazil. AIDS Care. 2013;25(6): 686–90. 10.1080/09540121.2013.764382 23394727

[52] Delicio AM, Lajos GJ, Amaral E, Cavichiolli F, Polydoro M, Milanez H. Adverse effects in children exposed to maternal HIV and antiretroviral therapy during pregnancy in Brazil: A cohort study. Reprod Health. 2018 5 10;15(1): 76 10.1186/s12978-018-0513-8 29747664 PMC5946413

[53] Wang L, Zhao H, Cai W, Tao J, Zhao Q, Sun L, et al Risk factors associated with preterm delivery and low delivery weight among HIV-exposed neonates in China. Int J Gynaecol Obstet. 2018 9;142(3): 300–307. 10.1002/ijgo.12532 29772068 PMC6103813

[54] Watts DH. Management of human immunodeficiency virus infection in pregnancy. N Engl J Med. 2002 6 13;346(24): 1879–91. 10.1056/NEJMra013338 12063373

[55] Thorne C, Rudin C, Newell M-L, et al Combination antiretroviral therapy and duration of pregnancy. AIDS 2000;14: 2913–20. 10.1097/00002030-200012220-00013 11398741

[56] Brocklehurst P, French R. The association between maternal HIV infection and perinatal outcome: A systematic review of the literature and meta-analysis. Br J Obstet Gynaecol. 1998;105(8): 836–848. 10.1111/j.1471-0528.1998.tb10227.x 9746375

[57] Mohammadi H, Papp E, Cahill L, Rennie M, Banko N, Pinnaduwage L, et al HIV antiretroviral exposure in pregnancy induces detrimental placenta vascular changes that are rescued by progesterone supplementation. Sci Rep. 2018 4 26;8(1):6552 10.1038/s41598-018-24680-w 29700323 PMC5919912

[58] Cameron N, Preece MA, Cole TJ. Catch-up growth or regression to the mean? Recovery from stunting revisited. Am J Hum Biol. 2005;17: 412–7. 10.1002/ajhb.20408 15981181

